# Mediators of the relationship between precarious employment and mental health

**DOI:** 10.1093/occmed/kqag006

**Published:** 2026-02-19

**Authors:** H Hasegawa, M Kizuki, S Okada

**Affiliations:** Department of Health Policy, Institute of Science Tokyo, Bunkyo, Tokyo 113-8519, Japan; Department of Public Health, Institute of Science Tokyo, Bunkyo, Tokyo 113-8519, Japan; Department of Health Policy, Institute of Science Tokyo, Bunkyo, Tokyo 113-8519, Japan

## Abstract

**Background:**

Precarious employment is associated with poor mental health among workers. However, the underlying mechanisms are not well understood.

**Aims:**

To investigate whether skill use and discretion, social environment, work intensity and working time quality mediate the relationship between precarious employment and mental well-being.

**Methods:**

Using the Sixth European Working Conditions Survey, data across 35 countries were analysed. We conducted causal mediation analyses to decompose the association between the precarious employment index and mental well-being (measured using the 5-item World Health Organization Well-Being Index [WHO-5]) into natural direct and indirect effects through four potential mediators, namely skill use and discretion, social environment, work intensity and working time quality. We additionally fitted a parallel multiple-mediator structural equation model as a robustness check.

**Results:**

Data from 31 903 employees were analysed. The total effect of the precarious employment index on WHO-5 scores was statistically significant (*P* < 0.001). The natural indirect effects were statistically significant through skill use and discretion index (34% mediated; 95% confidence interval, 28–40%) and social environment index (53% mediated; 95% confidence interval, 45–60%). Indirect effects through work intensity and working time quality were not statistically significant (both *P* > 0.05). The structural equation model showed the same pattern.

**Conclusions:**

Precarious employment negatively impacts skill use and discretion as well as social environment, which significantly mediate its effect on mental well-being. Interventions targeting these mediators may help mitigate the adverse effects of precarious employment on workers’ mental health.

Key learning points **What is already known about this subject:**

- Precarious employment, defined as employment insecurity, income inadequacy, and a lack of rights and protections, has emerged as a critical occupational health concern in contemporary society.
- The adverse impact of precarious employment on mental health is well-established in the literature.
- The mediating roles of specific job quality dimensions, such as skill use and discretion, social environment, work intensity and working time quality, in the relationship between precarious employment and mental health remain insufficiently understood.

**What this study adds:**

- Precarious employment adversely affects skill use and discretion as well as the social environment, which significantly mediate its negative impact on mental well-being.
- Work intensity and working time quality showed little evidence of mediating the association between precarious employment and mental well-being.
- Enhancing opportunities for skill use and discretion and strengthening workplace social support should be targeted strategies for mitigating the mental health consequences associated with precarious employment.

**What impact this may have on practice or policy:**

- Workers in precarious employment are at heightened risk for reduced mental well-being.
- Improving job quality, particularly skill use and discretion as well as social environment in the workplace, represents a relevant policy strategy for maintaining mental well-being among the growing population of precariously employed workers.
- Occupational health assessments may incorporate evaluations of skill use and discretion as well as social environment to guide strategies to prevent mental health problems.

## INTRODUCTION

Precarious employment, a form of low-quality work usually characterized by employment insecurity, income inadequacy, and a lack of rights and protections [1], is increasing globally [2]. At the population level, a repeated cross-sectional study in Sweden reported that the proportion of individuals who experienced precarious employment increased between 1992 and 2017 [3]. Similarly, at the individual level, a longitudinal analysis of a civilian cohort in the USA found a growing trend in employment precarity between 1988 and 2016 [4]. Precarious employment tends to persist; in a UK cohort, 44% remained in precarious work for over 4 years [5]. Entry into precarious employment at a young age is associated with greater persistence of precarity [6].

Precarious employment is associated with various adverse social outcomes, including reduced participation in social activities owing to work-related demands, reluctance to discuss one’s employment situation, challenges in managing regular expenses and limited financial capacity for social engagement [7]. Health impacts are particularly salient. Workers in highly precarious jobs reported 0.4 more unhealthy days and 1.2 more days of activity limitation per month than those in secure employment [8].

Studies have consistently demonstrated the association between precarious employment and adverse mental health outcomes. A cohort study in Sweden showed that individuals who were precariously employed in early adulthood had an elevated risk for subsequent hospital admissions for mental health disorders compared with individuals in standard employment relationships [9]. Similarly, panel data analysis of employed Australians showed that increases in employment precarity were associated with heightened depressive symptoms [10]. Additionally, a systematic review reported that persistent precarious employment lasting 12 months or longer was linked to poor mental health outcomes, including depressive symptoms [11]. Analyses of the European Working Conditions Survey (EWCS) have consistently shown a significant relationship between precarious employment and poorer mental well-being [12–15]. For instance, an analysis of the EWCS in 2015 found that higher levels of employment precarity were associated with progressively worse mental health [13]. Thus, understanding workplace-related mediators of the relationship between precarious employment and mental well-being may inform the development of targeted workplace interventions aimed at mitigating the adverse mental health effects of precarious employment.

The core elements of job quality that influence workers’ health and well-being include earnings, job security and prospects, skill use and discretion, social and physical work environments, work intensity and working time quality [16]. Among these, earnings, along with job security and prospects, are central components of the concept of precarious employment rather than the outcomes influenced by it. Additionally, substantial variations in the physical work environment across different economic sectors and occupational groups have been well-documented [17]. The other job quality dimensions—skill use and discretion, social environment, work intensity and working time quality—may be affected by precarious employment. A study showed that precariously employed workers reported lower levels of skill utilization and discretion and experienced less supportive workplace environments than those in more secure employment [18]. Another study reported that individuals in precarious employment experienced high rates of workplace bullying and discrimination, as well as low levels of control and greater exposure to passive work [19]. Among hotel workers, those temporarily employed had less control over their working hours; however, they reported lower work intensity and shorter working hours than did their permanently employed counterparts [20]. In addition, analyses using the EWCS in 2015 reported that the association between precarious employment and poorer mental health was attenuated after adjustment for psychological demands, job control and social support [21]. However, the extent to which skill use and discretion, social environment, work intensity and working time quality mediate the relationship between precarious employment and mental well-being remains insufficiently understood [22].

Therefore, we investigated the relationship between precarious employment and mental well-being among workers and explored the mediating roles of skill use and discretion, social environment, work intensity and working time quality.

## METHODS

We analysed data from the EWCS [23] conducted by Eurofound. The EWCS was first conducted in 1991, and subsequently in 1995, 2000, 2005, 2010, 2015, 2021 and 2024. We used the dataset from the sixth survey in 2015, as it was the most recent openly available dataset for research containing all required variables; several variables necessary to construct the indices used in this study were not collected in the 2021 telephone survey, and dataset from the eighth survey had not been released at the time of analyses.

The Sixth EWCS was conducted between February and December 2015 in 35 countries, including the 28 EU states at the time and Albania, Montenegro, North Macedonia, Norway, Serbia, Switzerland and Turkey. The eligibility criteria for the survey included residents aged 15 years or older (16 or older in Bulgaria, Norway, Spain and the UK) who were employed during the survey. To be considered employed, individuals should have worked for pay or profit for at least 1 h during the week preceding the interview.

In most countries, the target sample size was 1000 individuals. However, to better reflect the larger workforce in populous countries, the target sample size was increased in certain nations. Eurofound also offered countries the option to expand their sample sizes. The sample selection was performed using multistage stratified random sampling.

Face-to-face interviews were conducted in the participants’ homes and included a comprehensive set of questions about their working conditions. Full details of the survey methodology are available online [24].

The dataset was obtained from the UK Data Service (https://ukdataservice.ac.uk/). The study protocol was approved by the Ethics Committee of the Institute of Science Tokyo (E2024-049).

The primary predictor variable was the precarious employment index, whereas the primary outcome variable was mental well-being. Four potential mediating variables were examined: skill use and discretion, social environment, work intensity and working time quality indices. These mediators were constructed according to the definitions outlined in the overview report of the EWCS [17]. Details of the coding scheme for each index are provided in the Supplementary Material (available as Supplementary data at *Occupational Medicine* Online).

We developed the precarious employment index based on the framework established in the systematic review by Kreshpaj *et al.* [1]. The index encompassed three core subdimensions, namely employment insecurity, income inadequacy, and lack of rights and protections. Employment insecurity was evaluated based on factors such as contractual relationship instability, contractual temporariness, underemployment and the presence of multiple jobs. Income inadequacy was assessed using income level and volatility measures. Lack of rights and protection was determined by the absence of unionization and workplace rights. Each of the nine indicators was coded as 0 or 1, and the total score was calculated to represent the overall index. The index was standardized on a scale of 0–100.

Mental well-being was assessed using the total score from the 5-item World Health Organization Well-Being Index (WHO-5). The validity of the WHO-5 as a reliable screening tool for depression is well-documented [25].

The skill use and discretion index comprised the four subdimensions: cognition, decision latitude, organizational participation and training, which were assessed using five, four, three and two items, respectively.

The social environment index consisted of the two subdimensions, adverse social behaviour and social support, which were assessed using seven and eight items, respectively.

The work intensity index comprised three subdimensions, namely quantitative demands, pace determinants and interdependency, and emotional demands, which were assessed using four, five and three items, respectively.

The working time quality index consisted of four components, namely flexibility, working time arrangements, duration and atypical working time, which were assessed using one, one, three and four items, respectively.

Each item was coded as 0 or 1 or scored on a scale between 0 and 1. Subdimension scores were calculated by summing the item scores and standardizing them to a scale of 0–100 points. The overall index was determined by averaging the subdimension scores.

Based on previous research on workers’ mental health and data availability, we included several potential confounders in the analyses. These confounders were sex (male or female), age group (<35, 35–49 and ≥50 years), country of origin (nationals or foreign origin/background), living with a spouse/partner (yes or no), living with children (yes or no), educational attainment (primary and lower secondary, upper secondary or tertiary), company ownership (private sector, public sector or other sector), economic sector, and the International Standard Classification of Occupations-08 category.

We conducted a series of mediation analyses to examine the mechanisms linking precarious employment with mental well-being.

First, we performed causal mediation analyses using the mediate command in Stata 18. The total effect (TE) of precarious employment on mental well-being was decomposed into the natural direct effect (NDE) and the natural indirect effect (NIE) through each mediator. Standardized *z*-scores were used for the precarious employment index, WHO-5 well-being score and each mediator. All models were specified as linear and adjusted for all potential confounders, with standard errors clustered at the country level to account for within-country correlation. The outcome model included an exposure–mediator interaction term. The proportion mediated was calculated as the ratio of the indirect to the total effect (NIE/TE).

Second, to assess the simultaneous and specific indirect effects of the four mediators, we constructed a parallel mediation model using structural equation modelling (SEM). The SEM allowed correlations among the residuals of the mediators and accounted for sampling weights and clustering by country.

In addition, we examined the direct and indirect paths of association using multilevel regression models. The association between the precarious employment index and each potential mediator was evaluated using multilevel regression models. Similarly, the association between the precarious employment index and the WHO-5 score was assessed using multilevel regression models with and without adjustments for the four mediating variables. All potential confounders were included in the model as categorical variables.

Data with missing values for the precarious employment index, WHO-5 score or the four potential mediating variables were excluded from the analysis. Missing information related to the confounding variables was handled using the missing indicator method. All statistical analyses were conducted using the Stata 18 software (StataCorp LLC, College Station, TX, USA).

## RESULTS

**Figure 1.**
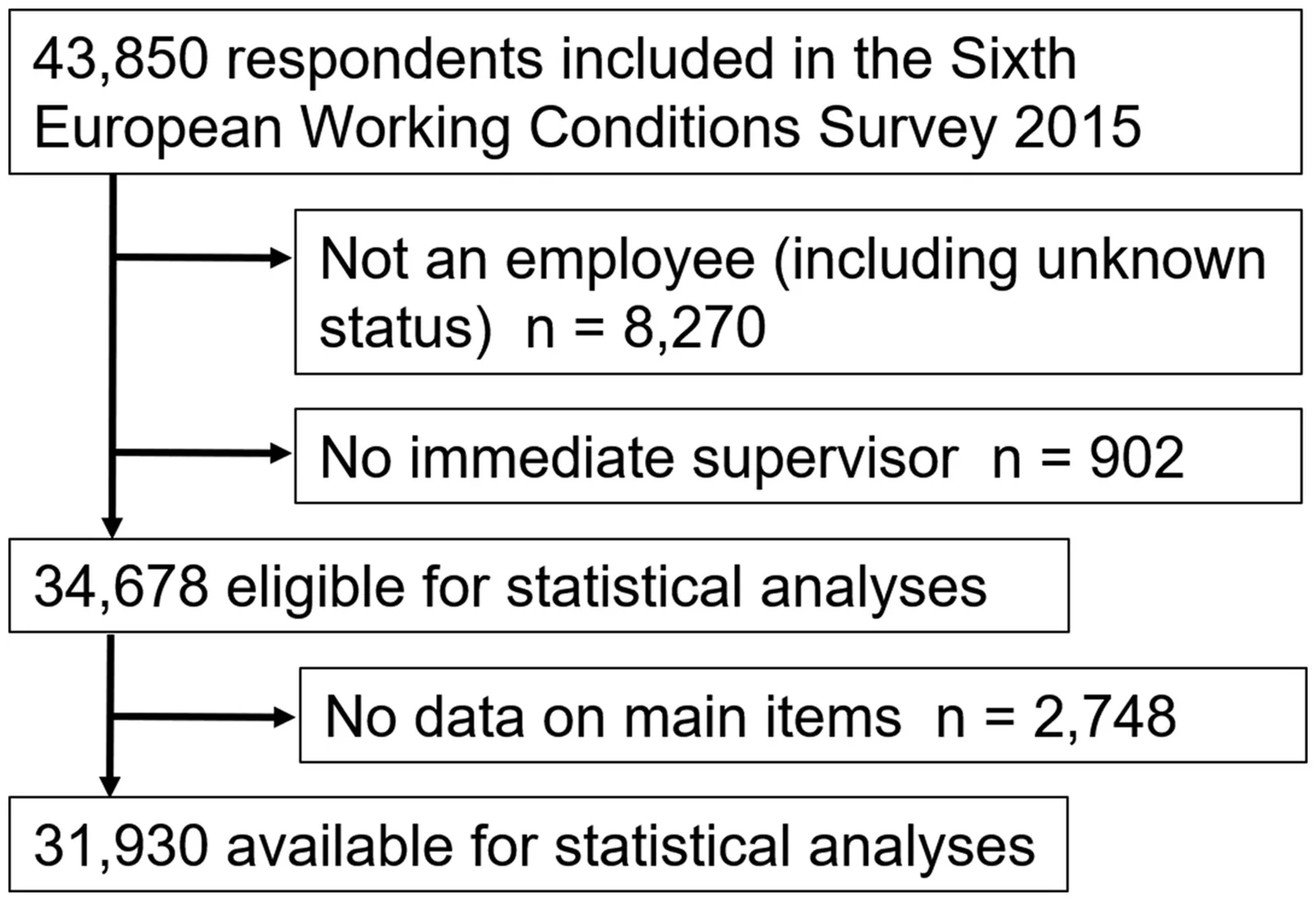
Flow of sample selection.

Figure 1 illustrates the sample selection process. Of the 43 850 workers included in the dataset, 8270 were excluded because they were not employees or had an unknown employment status, and an additional 902 were excluded because they did not have an immediate supervisor. Therefore, 34 678 participants were eligible for statistical analyses. Among these participants, 2748 (8%) had missing data for one or more of the following variables: the WHO-5 score (<1%) or precarious employment (<1%), skill use and discretion (2%), social environment (2%), work intensity (<1%) or working time quality (4%) index. Thus, the final sample size for statistical analyses was 31 903 participants. The sample sizes by country are listed in Table 1 (available as Supplementary data at *Occupational Medicine* Online).

Tables 1 and 2 summarize the descriptive statistics of the participants. Skewness values ranged from −0.85 to 0.69, and kurtosis values ranged from 2.3 to 3.7 for all continuous variables. These values are considered acceptable for assuming a normal distribution.

**Table 1.** Descriptive statistics of the participants (*N* = 31 903)

	***n*** (%)	Weighted %
Sex		
Male	15 354 (48)	52
Female	16 546 (52)	48
Missing	3 (0)	0
Age (years)		
Under 35	9525 (30)	33
35–49	12 523 (39)	39
50 and over	9780 (31)	28
Missing	75 (0)	0
Country of origin		
National origin	27 354 (86)	87
Foreign origin or background	4376 (14)	12
Missing	173 (1)	0
Living with a partner		
No	11 469 (36)	33
Yes	20 434 (64)	67
Living with children		
No	16 891 (53)	54
Yes	15 012 (47)	46
Educational attainment		
Primary and lower secondary	4881 (15)	17
Upper secondary	15 837 (50)	51
Tertiary	11 103 (35)	32
Missing	82 (0)	0
Company ownership		
Private sector	20 834 (65)	69
Public sector	9093 (29)	24
Other sector	1859 (6)	7
Missing	117 (0)	0
Economic sector		
Agriculture	570 (2)	2
Industry	5477 (17)	19
Construction	1952 (6)	6
Commerce and hospitality	6426 (20)	19
Transport	1869 (6)	6
Financial services	1184 (4)	4
Public administration	2160 (7)	7
Education	3239 (10)	9
Health	3584 (11)	12
Other	5313 (17)	16
Missing	129 (0)	0
Occupation		
Armed forces occupations	142 (0)	0
Managers	1538 (5)	4
Professionals	6269 (20)	19
Technicians and associate professionals	4104 (13)	15
Clerical support workers	3439 (11)	12
Service and sales workers	6865 (22)	19
Skilled agricultural, forestry and fish	310 (1)	1
Craft and related trades workers	3593 (11)	12
Plant and machine operators and assemblers	2427 (8)	8
Elementary occupations	3160 (10)	9
Missing	56 (0)	0

Weighted percentages were calculated using survey weights.

**Table 2.** Descriptive statistics for exposure, mediator and outcome variables (*N* = 31 903)

	Q1	Median	Q3	Mean	SD
Precarious employment index	11	22	33	20.8	16.0
Skill use and discretion index	33	52	70	51.8	24.2
Social environment index	75	88	100	83.8	17.4
Work intensity index	14	28	44	31.4	20.4
Working time quality index	54	67	75	65.4	18.9
WHO-5 score	56	72	80	68.1	20.1

Q1, 25th percentile; Q3, 75th percentile. The range of all indices is 0–100.

Table 3 presents the results of the causal mediation analysis. The TE of precarious employment on mental well-being was negative and statistically significant across all models. The NIEs through skill use and discretion and through social environment were both significant and in the same direction as the TE. The NIE through skill use and discretion accounted for 34% of the TE (95% confidence interval [CI] 28–40%), whereas that through social environment accounted for 53% (95% CI 45–60%). In contrast, the NIE through work intensity and working time quality were not significant and opposite in sign to the TE, suggesting little evidence of mediation through these variables.

**Table 3.** Causal mediation analysis of the association between precarious employment and mental well-being (*N* = 31 903)

	Estimate	Lower 95% CI	Upper 95% CI	
Skill use and discretion index				
Natural indirect effect	−0.090	−0.106	−0.073	***
Natural direct effect	−0.174	−0.209	−0.140	***
Total effect	−0.264	−0.301	−0.227	***
Proportion mediated (%)	34	28	40	***
Social environment index				
Natural indirect effect	−0.141	−0.157	−0.125	***
Natural direct effect	−0.127	−0.163	−0.091	***
Total effect	−0.269	−0.308	−0.229	***
Proportion mediated (%)	53	45	60	***
Work intensity index				
Natural indirect effect	0.003	−0.005	0.011	
Natural direct effect	−0.276	−0.314	−0.238	***
Total effect	−0.273	−0.312	−0.234	***
Proportion mediated (%)	—			
Working time quality index				
Natural indirect effect	0.004	−0.004	0.012	
Natural direct effect	−0.277	−0.316	−0.239	***
Total effect	−0.273	−0.312	−0.234	***
Proportion mediated (%)	—			

All models were estimated using the mediate command in Stata 18, with standardized variables for precarious employment, WHO-5 score and mediators. Linear models were adjusted for sex, age, country of origin, cohabitation with a partner, cohabitation with children, educational attainment, company ownership, economic sector, occupation and country (clustered standard errors at the country level). Effects are based on standardized variables and represent contrasts between −1 and +1 SD of the precarious employment index. Proportion mediated was calculated as NIE/TE × 100.

*** *P* < 0.001.

**Figure 2.**
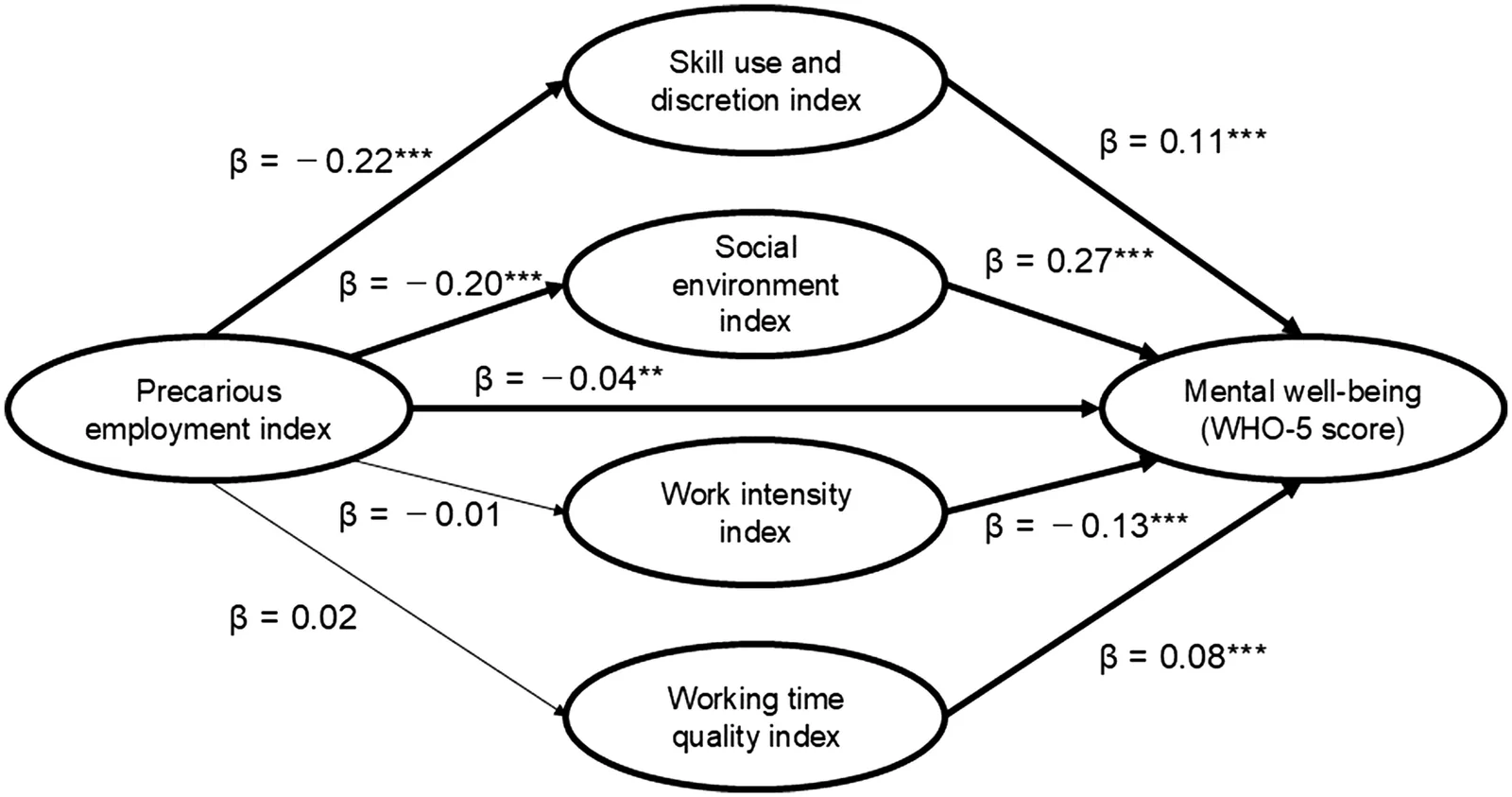
Structural equation model of precarious employment, job quality and mental well-being (*N* = 31 903). ***P < 0.001; **P < 0.01. Paths show standardized coefficients (i.e. the standard deviation change in the outcome per a 1 SD increase in the predictor) from a structural equation model. All variables depicted were standardized. Robust standard errors were clustered at the country level. Models were adjusted for sex, age, country of origin, cohabitation with a partner, cohabitation with children, educational attainment, company ownership, economic sector, occupation and country fixed effects. Survey weights were applied.

Figure 2 displays standardized path estimates from a parallel multiple-mediator SEM. The patterns of the SEM were consistent with those of the causal mediation analysis: precarious employment was associated with poorer mental well-being primarily through lower skill use and discretion and a poorer social environment. By contrast, the paths through work intensity and working time quality were not statistically significant. A small negative direct path from precarious employment to mental well-being remained after adjustment. The detailed results are presented in Table 2 (available as Supplementary data at *Occupational Medicine* Online).

The results for the associations between the precarious employment index and each mediator are reported in Tables 3–6 (available as Supplementary data at *Occupational Medicine* Online). The precarious employment index was significantly associated with low scores in the skill use and discretion (β = −0.32, *P* < 0.001) and social environment (β = −0.20, *P* < 0.001) indices but not with the work intensity or working time quality index (both *P* > 0.05).

Table 7 (available as Supplementary data at *Occupational Medicine* Online) provides the unadjusted associations, and Table 4 presents the adjusted relationships among the precarious employment index, four potential mediating variables and WHO-5 score. High indices of skill use and discretion, social environment and working time quality were significantly associated with high WHO-5 scores. Conversely, a high work intensity index was associated with a low WHO-5 score. The strength of the association between the precarious employment index and WHO-5 score was attenuated after adjusting for the four mediators, with the coefficient decreasing from β = −0.14 (*P* < 0.001) to β = −0.05 (*P* < 0.001).

**Table 4.** Multivariable multilevel regression models of mental well-being with and without mediators in the model (*N* = 31 903)

	Model 1 (without mediator)					Model 2 (with mediators)				
	β	Lower 95% CI	Upper 95% CI	*z*		β	Lower 95% CI	Upper 95% CI	*z*	
Precarious employment index	−0.14	−0.18	−0.10	−7.57	***	−0.05	−0.08	−0.02	−3.05	**
Skill use and discretion index						0.09	0.07	0.11	10.16	***
Social environment index						0.31	0.28	0.34	22.50	***
Work intensity index						−0.13	−0.16	−0.11	−9.40	***
Working time quality index						0.08	0.04	0.11	4.47	***
Sex										
Male	Reference					Reference				
Female	−2.41	−3.54	−1.28	−4.17	***	−2.87	−3.82	−1.93	−5.98	***
Missing	−14.52	−69.67	40.63	−0.52		−16.50	−61.46	28.46	−0.72	
Age (years)										
Under 35	Reference					Reference				
35–49	−3.03	−3.84	−2.21	−7.27	***	−2.26	−2.93	−1.59	−6.57	***
50 and over	−2.70	−3.83	−1.56	−4.66	***	−2.63	−3.63	−1.63	−5.15	***
Missing	−0.41	−4.32	3.51	−0.20		1.91	−0.73	4.55	1.42	
Country of origin										
National origin	Reference					Reference				
Foreign origin or background	0.25	−2.16	2.65	0.20		0.22	−1.73	2.17	0.22	
Missing	−9.92	−16.55	−3.28	−2.93	**	−8.63	−15.86	−1.41	−2.34	*
Living with a partner										
No	Reference					Reference				
Yes	0.46	−0.17	1.09	1.44		0.14	−0.44	0.73	0.48	
Living with children										
No	Reference					Reference				
Yes	−0.21	−1.04	0.61	−0.50		−0.24	−0.90	0.42	−0.72	
Educational attainment										
Primary and lower secondary	Reference					Reference				
Upper secondary	−0.46	−1.58	0.66	−0.80		−0.56	−1.54	0.42	−1.11	
Tertiary	−1.16	−2.31	−0.01	−1.98	*	−0.82	−1.55	−0.09	−2.21	*
Missing	7.54	0.22	14.86	2.02	*	7.95	0.32	15.58	2.04	*
Company ownership										
Private sector	Reference					Reference				
Public sector	−0.18	−1.41	1.05	−0.29		0.75	−0.45	1.94	1.22	
Other sector	0.16	−0.85	1.18	0.32		0.25	−0.43	0.92	0.72	
Missing	−2.24	−5.89	1.40	−1.21		−1.05	−3.80	1.70	−0.75	
Economic sector										
Agriculture	0.10	−3.35	3.55	0.06		−0.76	−4.58	3.06	−0.39	
Industry	−3.36	−4.53	−2.18	−5.61	***	−2.66	−3.87	−1.45	−4.30	***
Construction	−2.82	−4.52	−1.12	−3.25	**	−2.97	−4.59	−1.34	−3.57	***
Commerce and hospitality	Reference					Reference				
Transport	−1.46	−2.71	−0.21	−2.29	*	0.13	−1.13	1.40	0.21	
Financial services	0.16	−1.74	2.06	0.17		−1.00	−2.60	0.59	−1.23	
Public administration	−0.67	−2.71	1.37	−0.64		−1.84	−3.57	−0.10	−2.08	*
Education	2.05	−0.17	4.28	1.81		0.14	−1.71	2.00	0.15	
Health	−1.01	−2.50	0.48	−1.33		0.42	−1.35	2.20	0.47	
Other	0.49	−0.37	1.35	1.12		−0.78	−1.55	0.00	−1.97	*
Missing	−4.49	−6.50	−2.49	−4.39	***	−4.37	−7.91	−0.83	−2.42	*
Occupation										
Armed forces occupations	3.64	−1.35	8.63	1.43		1.22	−3.38	5.81	0.52	
Managers	0.34	−1.26	1.94	0.41		−2.45	−3.99	−0.91	−3.12	**
Professionals	1.07	−0.68	2.82	1.20		−1.21	−3.15	0.72	−1.23	
Technicians and associate professionals	1.77	0.63	2.91	3.05	**	−0.28	−1.37	0.81	−0.51	
Clerical support workers	1.24	−0.71	3.19	1.25		−0.76	−2.16	0.64	−1.06	
Service and sales workers	Reference					Reference				
Skilled agricultural, forestry and fish	4.27	0.19	8.35	2.05	*	2.77	−0.71	6.25	1.56	
Craft and related trades workers	1.60	−0.10	3.30	1.84		0.30	−1.08	1.68	0.43	
Plant and machine operators and assemblers	−0.64	−3.04	1.76	−0.52		0.51	−1.27	2.28	0.56	
Elementary occupations	−1.22	−3.45	1.02	−1.06		−1.18	−2.39	0.02	−1.92	
Missing	−0.28	−5.46	4.90	−0.10		−1.23	−5.02	2.56	−0.64	

Weighted multilevel regression analyses were performed for the WHO-5 score (range: 0–100), with country as a random effect. Model 1 incorporated all variables listed in this table, except for the skill use and discretion index, social environment index, work intensity index and working time quality index as independent variables, while Model 2 incorporated all variables listed in this table. The variance inflation factors for all fixed‐effect covariates were <5 when assessed in ordinary least squares versions of Model 1 and Model 2. Survey weights were applied to all estimates.

*** *P* < 0.001;

** *P* < 0.01;

* *P* < 0.05; β, partial regression coefficient.

## DISCUSSION

The analyses showed that precarious employment was adversely associated with mental well-being among European employees and that this association was largely explained by two job quality dimensions. TE was mainly mediated through social environment and to a lesser extent through skill use and discretion. By contrast, we found no clear evidence of mediation by work intensity and working time quality. These patterns were consistent with those in an SEM and were corroborated by multilevel regression analyses, indicating robustness across modelling approaches.

This study has several strengths. We leveraged a large, multicountry dataset (Sixth EWCS) specifically designed to assess working conditions across Europe and to examine interrelations among work-related factors and vulnerable groups [17]. Analytically, we prespecified mediators from an established job quality framework and conducted robustness checks using SEM and multilevel regression, which yielded results consistent with the causal mediation findings.

Limitations also warrant consideration. The cross-sectional design precludes causal inference and limits the temporal ordering of precarious employment, mediators and mental well-being. Accordingly, our findings should be interpreted as associative rather than causal. Although we adjusted for a broad set of sociodemographic and job-related factors, residual confounding is possible given data constraints. For example, ethnicity, language proficiency and marital status are associated with mental well-being [26, 27]. We used country of origin and cohabitation with a partner as proxies, may attenuate but cannot fully eliminate confounding. Moreover, adjustment for highest educational attainment does not fully eliminate confounding by prior academic performance. All constructs were self-reported, raising the possibility of common-method variance and reporting bias. Non-differential measurement error in continuous independent variables would generally attenuate associations towards the null, implying that the indirect effects are likely underestimated. By contrast, random error in the WHO-5 score primarily inflates variance and reduces precision in linear models. Finally, because the survey was conducted in Europe, generalizability to other regions may be limited.

This study’s finding that precarious employment is associated with reduced skill use and discretion, as well as a less supportive social environment in the workplace is consistent with the results of a previous cross-sectional analysis [18]. A scoping review also reported that precarious workers frequently experience marginalization in the workplace, marked by unpleasant tasks, insufficient training, limited access to professional development opportunities, and exclusion from information networks and decision-making processes [28]. By contrast, the association between precarious employment and work intensity was not statistically significant. This finding is consistent with a previous cross-sectional analysis [18]. Similarly, the absence of an association between precarious employment and work intensity in the present study aligns with the findings of a Swedish study, which reported no link between precarious employment and high work demands [19]. Nevertheless, as noted in the limitations, potential measurement error means that a null mediation estimate should not be taken as definitive evidence of no mediation.

From a public health perspective, workers’ mental health is a major concern, affecting 84 million individuals across the EU states and imposing an economic burden exceeding 4% of the region’s gross domestic product [29]. The present study confirmed that, high skill use and discretion, social environment and working time quality indices were associated with good mental well-being; a high work intensity index was associated with poor mental well-being. These mediating variables were originally developed to capture the dimensions of job quality relevant to mental well-being [16], and the present findings further support the construct validity of these indices. Therefore, regardless of their association with precarious employment, these variables warrant consideration in efforts to promote mentally healthy workplace environments.

The present analysis suggests that precarious employment also influences mental well-being through pathways that are not fully mediated by the workplace environment. A qualitative study showed that workers in precarious employment experience heightened emotional stress and depressive symptoms stemming from employment insecurity, income instability, and a reduced capacity to maintain health and overall well-being [30]. Additionally, financial strain, temporal uncertainty, marginal status and employment insecurity have been identified as key contributors to poor mental health among precarious workers [28]. Furthermore, poor sleep quality partially mediates the relationship between precarious employment and mental health [31]. The presence of a direct path suggests the significance of considering psychosocial and physiological pathways in addressing these effects.

Our findings refine prior evidence by using broad job quality indices and formal mediation analysis to show that the association between precarious employment and mental well-being operates chiefly through the skill use and discretion and social environment. Future work may clarify the temporal dynamics of this pathway by tracking changes in exposure, mediators and outcomes over time. Longitudinal designs with repeated measures and richer covariates can reduce bias. Extending analyses beyond Europe can also enhance generalizability.

In conclusion, precarious employment negatively impacts skill use and discretion as well as social environment, which significantly mediate its effect on mental well-being. Policies and organizational practices targeting these mediators, i.e. expanding decision latitude, participation, training and social support at work, may help mitigate the adverse effects of precarious employment on workers’ mental health.

## Supplementary Material

kqag006_Supplementary_Data
