# Supplementary material for: Mediators of the relationship between precarious employment and mental health
Source: Occup Med (Lond). 2026 Feb 19;76(2):108–16. doi: 10.1093/occmed/kqag006 (PMC13095213; doi:10.1093/occmed/kqag006)
Supplement: kqag006_Supplementary_Data [file kqag006_supplementary_data.zip › kqag006_Supplementary_Data/Supplementary material.docx]

**Supplementary material**

**Survey questions and data coding methods**

The survey questions were sourced from the EWCS 2015 Master Questionnaire (https://www.eurofound.europa.eu/en/surveys-and-data/surveys/european-working-conditions-survey/ewcs-2015).

***Precarious employment***

1. Employment insecurity
   1. Contractual relationship insecurity

Q89. To what extent do you agree or disagree with the following statements about your job?

G) I might lose my job in the next 6 months.

1 – Strongly agree/Tend to agree

0 – Neither agree nor disagree/Tend to disagree/Strongly disagree/Not applicable/Don’t know

- 1. Contractual temporariness

Q11. What kind of employment contract do you have in your main job?

1 – Contract of limited duration/A temporary employment agency contract/An apprenticeship or other training scheme/No contract

0 – Contract of unlimited duration

- 1. Contractual underemployment

Q24. How many hours do you usually work per week in your main paid job?

1 – Less than 35 hours

0 – 35 hours and more

- 1. Multiple jobs

Q27. Besides your main paid job, do you have any other paid job(s)?

1 – Yes, regular/Yes, occasional

0 – No other paid job

1. Income inadequacy
   1. Income level

Q104. Please can you tell us how much are your NET monthly earnings from your main paid job?

1 – Below the 20th percentile (PPP-adjusted)

0 – At or above the 20th percentile (PPP-adjusted)

- 1. Income volatility

Q18. Now I would like you to think about the last 12 months. During the last 12 months, has your work changed in any of the following ways?

B) Your salary or income

1 – Decreased a lot

0 – Increased a lot/Increased a little/No change/Decreased a little/Not applicable/Don’t know

1. Lack of rights and protection
   1. Lack of unionisation

Q71. Does the following exist at your company or organisation…?

A) Trade union, works council, or a similar committee representing employees

1 – No

0 – Yes

C) A regular meeting in which employees can express their views about what is happening in the organisation

1 – No

0 – Yes

- 1. Lack of workplace rights

Q33. Regarding the health and safety risks related to the performance of your job, how well informed would you say you are?

F) You can take a break when you wish.

1 – Not very well informed/Not at all well informed/Don’t know

0 – Very well informed/Well informed

***Skill use and discretion***

1. Cognitive dimension

Q53. Generally, does your main paid job involve...

C) solving unforeseen problems on your own

1 – Yes

0 – No

E) complex tasks

1 – Yes

0 – No

F) learning new things

1 – Yes

0 – No

Q30. Please tell me, using the same scale, does your main paid job involve...?

I) working with computers, laptops, smartphones, etc.

1 – All of the time/Almost all of the time/Around three-quarters of the time/Around half of the time/Around one-quarter of the time

0 – Almost never

Q61. For each of the following statements, please select the response which best describes your work situation.

I) You are able to apply your own ideas in your work.

1 – Always/Most of the time

0 – Sometimes/Rarely/Never

1. Decision latitude

Q54. Are you able to choose or change...

A) your order of tasks?

1 – Yes

0 – No

B) your methods of work?

1 – Yes

0 – No

C) your speed or rate of work?

1 – Yes

0 – No

Q61. For each of the following statements, please select the response which best describes your work situation.

E) You have a say in the choice of your work colleagues.

1 – Always/Most of the time

0 – Sometimes/Rarely/Never

1. Organisational participation

Q61. For each of the following statements, please select the response which best describes your work situation.

C) You are consulted before objectives are set for your work.

1 – Always/Most of the time

0 – Sometimes/Rarely/Never

D) You are involved in improving the work organisation or work processes of your department or organisation.

1 – Always/Most of the time

0 – Sometimes/Rarely/Never

N) You can influence decisions that are important for your work.

1 – Always/Most of the time

0 – Sometimes/Rarely/Never

1. Training

Q65. Over the past 12 months, have you undergone any of the following types of training to improve your skills?

A) Training paid for or provided by your employer

1 – Yes

0 – No/Don’t know

C) On-the-job training (co-workers, supervisors)

1 – Yes

0 – No/Don’t know

***Social environment index***

1. Adverse social behaviour

Q80. Over the last month, during the course of your work, have you been subjected to any of the following?

A) Verbal abuse

1 – Yes

0 – No

B) Unwanted sexual attention

1 – Yes

0 – No

C) Threats

1 – Yes

0 – No

D) Humiliating behaviours

1 – Yes

0 – No

Q81. Over the past 12 months, during the course of your work, have you been subjected to any of the following?

A) Physical violence

1 – Yes

0 – No

B) Sexual harassment

1 – Yes

0 – No

C) Bullying/harassment

1 – Yes

0 – No

1. Social support

Q63. To what extent do you agree or disagree with the following statements? Your immediate boss…

A) respects you as a person.

1 – Strongly agree/Tend to agree

0 – Neither agree nor disagree/Tend to disagree

B) gives you praise and recognition when you do a good job.

1 – Strongly agree/Tend to agree

0 – Neither agree nor disagree/Tend to disagree

C) is successful in getting people to work together.

1 – Strongly agree/Tend to agree

0 – Neither agree nor disagree/Tend to disagree

D) is helpful in getting the job done.

1 – Strongly agree/Tend to agree

0 – Neither agree nor disagree/Tend to disagree

E) provides useful feedback on your work.

1 – Strongly agree/Tend to agree

0 – Neither agree nor disagree/Tend to disagree

F) encourages and supports your development.

1 – Strongly agree/Tend to agree

0 – Neither agree nor disagree/Tend to disagree

Q61. For each of the following statements, please select the response which best describes your work situation.

A) Your colleagues help and support you.

1 – Always/Most of the time

0 – Sometimes/Rarely/Never

B) Your manager helps and supports you.

1 – Always/Most of the time

0 – Sometimes/Rarely/Never

***Work intensity index***

1. Quantitative demands

Q49. Does your job involve…

A) working at very high speed?

1 – All of the time/Almost all of the time/Around three-quarters of the time

0 – Around half of the time/Around one-quarter of the time/Almost never/Never

B) working to tight deadlines?

1 – All of the time/Almost all of the time/Around three-quarters of the time

0 – Around half of the time/Around one-quarter of the time/Almost never/Never

Q61. For each of the following statements, please select the response which best describes your work situation.

G) You have enough time to get the job done.

1 – Rarely/Never

0 – Always/Most of the time/Sometimes

Q51. How often do you have to interrupt a task you are doing in order to take on an unforeseen task?

1 – Very often/Fairly often

0 – Occasionally/Never

1. Pace determinants and interdependency
2. Pace determinants

Q50. On the whole, is your pace of work dependent on…

A) the work done by colleagues?

1 – Yes

0 – No

B) direct demands from people such as customers, passengers, pupils, patients, etc?

1 – Yes

0 – No

C) numerical production targets or performance targets?

1 – Yes

0 – No

D) automatic speed of a machine or movement of a product?

1 – Yes

0 – No

E) the direct control of your boss?

1 – Yes

0 – No

1. Interdependency

1 – Three or more out of five pace determinants

0 – Two or fewer out of five pace determinants

1. Emotional demands

Q61. For each of the following statements, please select the response which best describes your work situation.

O) Your job requires that you hide your feelings.

1 – Always/Most of the time

0 – Sometimes/Rarely/Never

Q30. Please tell me, using the same scale, does your main paid job involve…

G) handling angry clients, customers, patients, pupils, etc?

1 – All of the time/Almost all of the time/Around three-quarters of the time

0 – Around half of the time/Around one-quarter of the time/Almost never/Never

H) being in situations that are emotionally disturbing for you?

1 – All of the time/Almost all of the time/Around three-quarters of the time/Around half of the time/Around one-quarter of the time

0 – Almost never/Never

***Working time quality***

1. Duration (reverse-scored item)

Q24. How many hours do you usually work per week in your main paid job?

1 – 48 hours or more per week

0 – Between 1 and 47 hours per week

Q38. In the last month, has it happened at least once that you had less than 11 hours between the end of one working day and the start of the next working day?

1 – Yes

0 – No

Q37. Normally, how many times a month do you work…

D) more than 10 hours a day?

1 – At least once (1 to 31 days)

0 – Never

1. Atypical working time

Q37. Normally, how many times a month do you work…

A) at night, for at least 2 hours between 10.00 pm and 5.00 am?

1 – Never

0 – At least once (1 to 31 days)

B) on Sundays?

1 – Never

0 – At least once (1 to 5 days)

C) on Saturdays?

1 – Never

0 – At least once (1 to 5 days)

Shift work

Q39. Do you work...

E) shifts?

Q41. Do you work...?

1 – No shift

0.66 – Permanent shifts

0.33 – Alternating shifts

0 – Daily split shifts

1. Working time arrangements

Q42. How are your working time arrangements set?

Q43. Do changes to your working time arrangements occur regularly? (IF YES) How long before are you informed about these changes?

1 – Working time arrangement is not set by the company or set by the company, but no changes in arrangements occur

0.75 – Working time arrangement is set by the company, and changes occur several weeks in advance

0.5 – Working time arrangement is set by the company, and changes occur several days in advance

0.25 – Working time arrangement is set by the company, and changes occur the day before

0 – Working time arrangement is set by the company, and changes occur on the same day

1. Flexibility

Q47. Would you say that for you, arranging to take an hour or two off during working hours to take care of personal or family matters is...

1 – Very easy

0 – Fairly easy/Fairly difficult/Very difficult
